# Ruxolitinib in steroid refractory graft-vs.-host disease: a case report

**DOI:** 10.1186/s13045-016-0298-6

**Published:** 2016-08-08

**Authors:** Enrico Maffini, Luisa Giaccone, Moreno Festuccia, Lucia Brunello, Ilaria Buondonno, Dario Ferrero, Mario Boccadoro, Chiara Dellacasa, Alessandro Busca, Domenico Novero, Benedetto Bruno

**Affiliations:** 1 Department of Oncology, SSCVD Trapianto di Cellule Staminali, A.O.U. Città della Salute e della Scienza di Torino, Torino, Italy; 2 Department of Molecular Biotechnology and Health Sciences, University of Torino, Torino, Italy; 3 Department of Oncology, University of Torino, Torino, Italy; 4 Department of Pathology, AOU Città della Salute e della Scienza di Torino, University of Torino, Torino, Italy

**Keywords:** Allogeneic hematopoietic stem cell transplant (HSCT), Steroid-refractory graft-vs.-host disease (SR-GvHD), Ruxolitinib, Regulatory T cells (Treg), Proinflammatory cytokines, Case report

## Abstract

**Background:**

Allogeneic hematopoietic stem cell transplantation (HSCT) is potentially curative in a variety of hematological malignancies. Graft-vs.-host disease (GvHD) remains a life-threatening complication. Standard treatment is high-dose (HD) corticosteroids. Steroid-refractory (SR) GvHD is associated with poor prognosis. At present, second-line treatment is ill-defined and includes a number of agents. Novel insights into the pathophysiology of acute GvHD (aGvHD) highlight the relevant role of the host inflammatory response governed by several kinase families, including Janus kinases (JAK)1/2. Ruxolitinib, a JAK1/2 inhibitor approved for intermediate-2/high-risk myelofibrosis, was recently employed in SR-GvHD with encouraging overall response rates. Clinical experience however remains limited.

**Case presentation:**

A 51-year-old male with refractory anemia with excess blast type-2 underwent a myeloablative allogeneic HSCT from a 9/10 HLA-matched unrelated donor after conditioning with busulfan and cyclophosphamide. GvHD prophylaxis consisted of cyclosporine, methotrexate, and thymoglobulin. CD34^+^ cells/kg infused were 8.69 × 10^6^ kg. On day 29, the patient developed overall grade IV aGvHD with biopsy proven stage IV gastrointestinal (GI) GvHD refractory to HD corticosteroids. Patient conditions rapidly deteriorated and became critical despite the addition of mycophenolate mofetil and budesonide. On day 33, Ruxolitinib was started, and on day 39 the patient clinical conditions gradually improved. Complete resolution of aGvHD was also confirmed by histology on day 54.

**Conclusions:**

At 5 months from HSCT, the patient is well and in continuous hematological complete remission without flare of GvHD. Ruxolitinib was discontinued on day 156. Ruxolitinib is feasible and effective in SR-aGvHD though large prospective clinical trials are warranted.

## Background

Allogeneic hematopoietic stem cell transplantation (HSCT) is potentially curative for several hematopoietic malignancies. Acute graft-vs.-host disease (aGvHD) remains the major cause of morbidity and mortality. High-dose corticosteroids (methylprednisolone, 1–2 mg/kg per day) are currently considered standard first-line treatment. However, a remarkable number of patients do not respond [1]. Estimated incidence of steroid-refractory aGvHD (SR-GvHD) is some 40 %, and it is associated with poor long-term survival of 5–30 % [2–4]. At present, there are no well-defined treatment approaches although several second-line therapies aimed at inactivating alloreactive donor T cells, pro-inflammatory cytokines or their receptors have been investigated. The weighted average 6-month survival from 25 retrospective studies or phase II trials was 49 % [2]. Among others, agents used included anti-thymocyte globulin, mycophenolate mofetil, anti-CD25 monoclonal antibody (basiliximab), anti-tumor necrosis factor alpha (infliximab and etanercept), anti-interleukin 2 receptor-alpha (inolimomab), anti-CD52 (alemtuzumab), pentostatin, m-Tor inhibitors and extracorporeal photoapheresis [5–13]. Since clinical trials in SR-GvHD are difficult to design given the heterogeneity of treatment policies, direct comparisons of different agents have not been possible.

## Case presentation

**Fig. 1:**
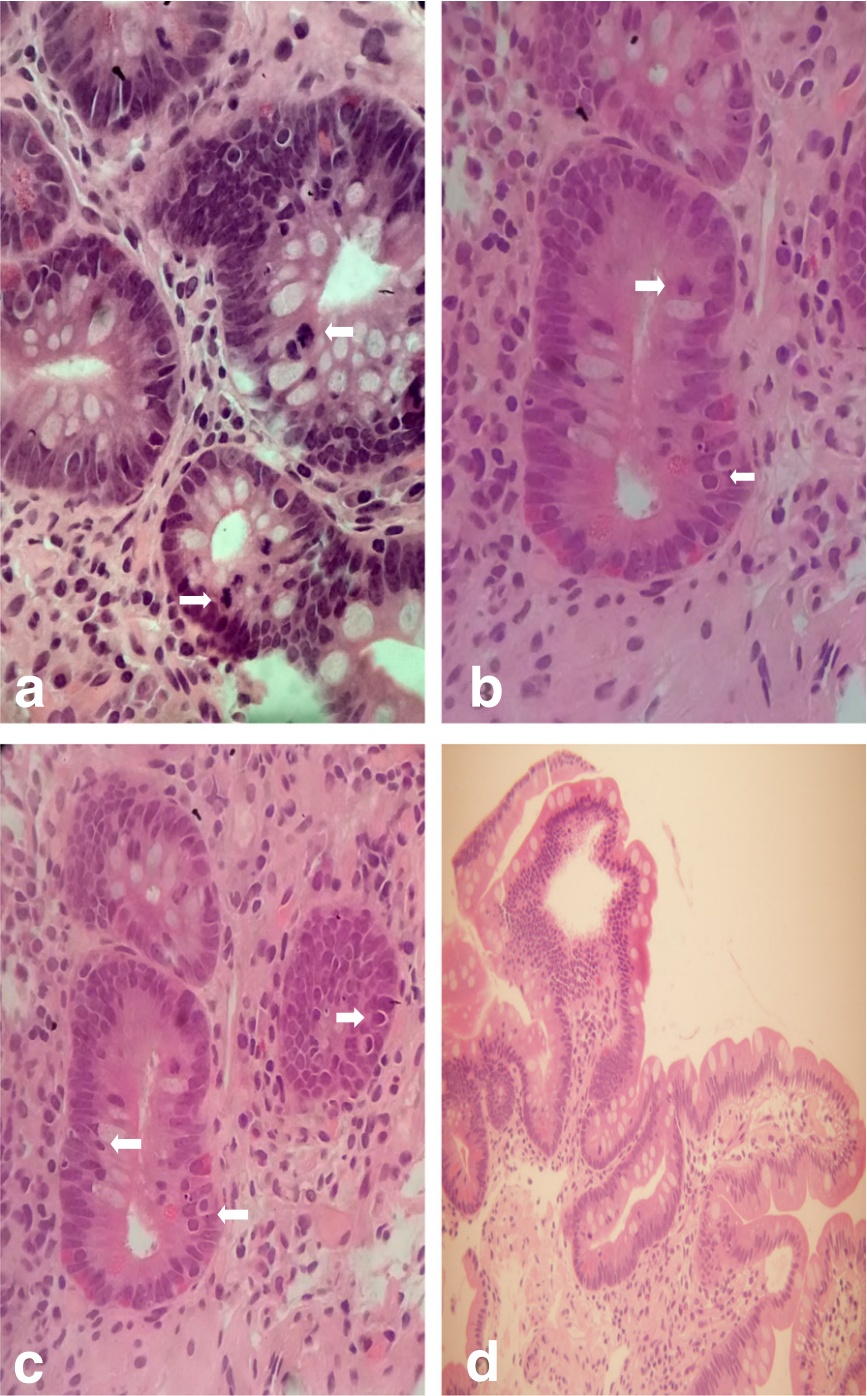
Histology studies (H&E). **a**–**c** Gastrointestinal acute grade I GVHD: focal apoptosis of crypt epithelial cells (*white arrows*) without abscess or crypt destruction. A clear lymphocytic infiltration of the lamina propria is not present. Moderate mucosal atrophy can be observed. **d** Duodenum at day +54 post-transplant: complete reconstitution of the mucosa with disappearance of apoptotic crypt cells (courtesy of D. Novero, Pathology, University of Turin)

A 51-year-old male without significant comorbidities and irrelevant past medical history was diagnosed with refractory anemia with excess blast type-1 (RAEB-1) in 2013 and evolved to RAEB-2 after 24 months. Overall, the patient had a high risk score both by the International (IPSS) and by the WHO prognostic scoring systems (WPSS). Blast count was 15 % on bone marrow biopsy and 11 % on marrow aspirate. Other features included trisomy 8 by cytogenetic analysis and two cytopenias—neutropenia and thrombocytopenia—in the peripheral blood. He never required red blood cell or platelet transfusions. He was initially treated with 2 cycles of induction chemotherapy with fludarabine (30 mg/m^2^, days 1–5), high-dose cytarabine (2000 mg/m^2^, days 1–5), and idarubicin (10 mg/m^2^, days 1–3). Complete remission by histology and by flow cytometry was obtained. However, FISH analysis showed persistent trisomy 8. The patient underwent an allogeneic HSCT with mobilized peripheral blood stem cells from a 9/10 HLA-matched (antigenic mismatch at HLA-A) unrelated donor. Conditioning regimen was busulfan (3.2 mg/kg/day; days −7 to −4) and cyclophosphamide (60 mg/kg/day; days −3 and −2). GvHD prophylaxis consisted of cyclosporine (CsA) (1.5 mg/kg twice daily, from day −1), methotrexate (15 mg/m^2^, 24 h after transplant, then 10 mg/m^2^ on days 3, 6, and 11), and thymoglobulin (2.5 mg/kg on days −3 and −2). CD34^+^ cells/kg infused were 8.69 × 10^6^. Gut decontamination with antibiotics after HSCT was not scheduled. On day 13, the patient stopped oral intake because of grade III mucositis. On day 22, he developed a maculo-papular rash on 50 % of his body surface area (stage 2) and mild (500 ml/24 h) watery diarrhea (stage 1), without fever and/or liver function tests abnormalities suggestive of aGVHD. Stool cultures ruled out gastrointestinal (GI) bacterial, viral or parasitic infections. A chest X-ray ruled out pulmonary infiltrates. Diagnosis of overall grade II aGVHD was made and intravenous (i.v.) corticosteroids at 2 mg/kg/day were promptly started on the same day. However, diarrhea worsened rapidly over the following days to stage 4 GI aGvHD with over 2500 mL/24 h of diarrhea and increasing painful abdominal cramps (Table 1). Clinical conditions did not improve despite the combination of cyclosporine, high-dose steroids and the addition of oral budesonide (3 mg three times per day) on day 28 and i.v. mycophenolate mofetil (MMF) (1 g three times per day) on day 29. An endoscopic evaluation of the upper GI tract with multiple biopsies on day 33 confirmed the diagnosis of aGvHD (Fig. 1a–c). Patient's clinical conditions rapidly deteriorated. Oral Ruxolitinib was started at 5 mg twice per day on day 33. Stools volume progressively and steadily decreased to less than 1000 mL on day 39. MMF was stopped on the same day given its unlikely clinical efficacy and to reduce immunosuppression. Clinical conditions gradually improved. Neutrophil and platelet engraftment (defined as the first of 3 consecutive days of neutrophils ≥500/uL and as the first of 7 consecutive days with platelet counts ≥20,000/uL without transfusion support, respectively) occurred at days 17 and 19. Platelet counts were ≥300.000/uL on day 28 and then dropped below 100,000/uL on day 49 and remained stable around 40,000–50,000/uL until discharge (with concurrent oral Ruxolitinib at 5 mg twice daily). Hemoglobin values peaked at 136 g/L on day 31 and dropped below 100 g/L on day 35. Overall, 4 units of red blood cells were required during hospitalization. Skin lesions disappeared completely by day 50. On day 45, the patient resumed oral food intake without nausea and/or vomiting and by day 61 stools were formed. A second endoscopy with multiple biopsies on day 54 revealed no residual signs of aGvHD in the GI tract (Fig. 1d). Bone marrow biopsy on day 64 showed a hypocellular marrow with normal myeloid maturation without evidence of disease recurrence and full donor engraftment by mixed-chimerism analysis. On day 70, the patient was discharged. Ruxolitinib was initially reduced because of progressive pancytopenia to 5 mg per day on day 100 and to 5 mg every other day on day 107. It was resumed at 5 mg per day on day 113 because of soften stools and abdominal discomfort with prompt improvement of GI symptoms. Platelet counts progressively raised to ≥80,000/uL.

**Table 1:** Patient timeline clinical history

Days from HSCT	Clinical condition/therapeutic intervention
0	HSCT
17	Neutrophil recovery
19	Platelet recovery
22	aGvHD onset: diarrhea (st.I) and skin (st.II). Started PDN-equivalent 2 mg/kg/iv
28	Diarrhea exceeded 1500 mL/day (st.III) and started budesonide 3 mg tid po
29	Diarrhea exceeded 2000 mL/day (st.IV) and started MMF 1 g tid iv
33	EGDS with biopsies (GvHD confirmation). Started Ruxolitinib 5 mg bid
36	Steroid taper
39	Diarrhea below 1000 mL and Ruxolitinib 5 + 10 mg/day. Stop MMF
45	Resumed oral food intake
49	Switch to oral CsA
54	EGDS with biopsies: no signs of GvHD
61	Switch PDN po
66	Reduced Ruxolitinib to 5 mg bid
70	Patient discharged
100	Reduced Ruxolitinib to 5 mg/day
135	Steroid stopped
156	Ruxolitinib stopped

*abbreviations*: *HSCT* hematopoietic stem cell transplantation, *aGvHD* acute graft-vs.-host disease, *PDN* prednisone, *iv* intravenously, *po* orally, *MMF* mycophenolic acid, *EGDS* esophagous-gastro-duodenoscopy, *CsA* cyclosporine A, *bid* twice daily, *tid* three times a day, *st* stage

### Cytokine measurement

**Fig. 2:**
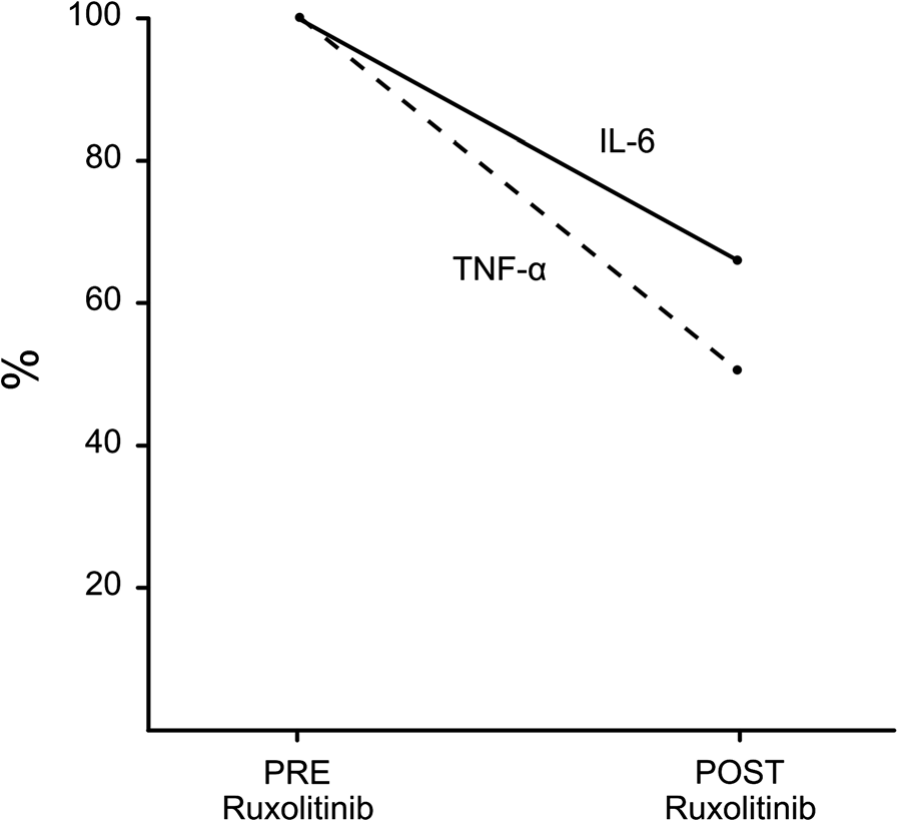
Serum levels of TNF-α and IL-6. TNF-α decreased from 10 pg/mL at day 27 (while on high-dose steroids) to 5 pg/mL at day 54, while IL-6 decreased from 2.3 to 1.5 pg/mL (values are expressed as percentage)

Serum levels of tumor necrosis factor alpha (TNF-α) and interleukin-6 (IL-6) were measured in serum samples from day +27 (before the start of Ruxolitinib) and day +56 (after 23 days of treatment), by Becton Dickinson Biosciences Human Inflammatory Cytokine kit. Interestingly, we observed a decrease of both these pro-inflammatory markers during Ruxolitinib treatment (Fig. 2). We cannot, however, rule out a potential effect of glucocorticoids and mycophenolic acid [14, 15].

## Conclusions

Donor T cell immune response to recipient antigens represents a key mechanism of GvHD and is combined with a massive production of inflammatory cytokines such as TNF-α, IL-1, IL-6 and IL-2-R. Cytokine-induced activation of the various effector cells—including T cells, dendritic cells, and neutrophils—is mediated by the interplay between cytokine receptors and a number of specialized kinases [16]. Family members of Janus kinases (JAK) are among the most studied. Ruxolitinib is a selective oral JAK1/2 inhibitor approved for the treatment of patients with intermediate-2 or high-risk primary myelofibrosis [17, 18]. Spleen size reduction and improvement of constitutional symptoms are directly correlated with inhibition of JAK-STAT signal transducer hyperactivity and with reduction of both effector cells activity and flogistic cytokine signature [19]. Apart from its ability to depress the pro-inflammatory environment of GvHD, Ruxolitinib shapes T cell mediated immune response toward a FoxP3^+^ regulatory T cell (Treg) polarization mostly by a sparing mechanism of the JAK3-STAT5 pathway [18]. Tregs improve signs and symptoms of aGvHD and promote immunotolerance [19–23]. These mechanisms have been shown on murine models [14] and also form the rationale for the potential efficacy of Ruxolitinib in the treatment of GvHD in man.

Only a few clinical experiences have been reported so far. Spoerl et al. [24] explored JAK1/2 inhibition during aGvHD both in a major HLA-mismatched murine model and in a cohort of 6 HSCT recipients who developed SR-aGvHD (GI in 2 and skin in 4). In the murine model, Ruxolitinib was associated with a significantly prolonged survival with reduced weight loss and GvHD severity by histology studies and suppression of the serum inflammatory cytokine profile. Moreover, a reduction of donor alloreactive T cells with a concomitant expansion of CD4^+^ FoxP3^+^ Treg in GvHD target organs demonstrated the ability of Ruxolitinib to shift T cell phenotype. In HSCT recipients, response rates were optimal with clinical regression in all and a marked reduction of pro-inflammatory cytokines such as IL-6 and soluble IL-2 receptor. Ruxolitinib was employed at a starting dose of 5 mg twice per day daily with a dose increase to 10 mg twice per day. Neither thrombocytopenia nor anemia were reported. A recent retrospective multi-center survey on 95 HSCT recipients with SR-GVHD—54 acute and 51 chronic GvHD (cGvHD), reported the most significant clinical experience so far completed [25]. Median prior lines of treatment were three (range: 1–7 for aGvHD; 1–10 for cGvHD). Overall response rate was 81.5 % in the aGvHD group (44/54) with 57 % (25/44) complete remissions and of 85.4 % (35/41) in the cGvHD group, respectively. Overall survival was 79 and 97.4 %. Among responders, GvHD flare was 6.8 % for aGvHD and 5.7 % for cGvHD patients. Side effects such as cytopenia (55.6 and 17.1 % in the aGvHD and cGvHD group, respectively) and cytomegalovirus (CMV) reactivation (33.3 and 14.6 %) were comparable to those reported with other agents including steroids, infliximab, alemtuzumab, mycophenolate mofetil, or cyclosporine. Overall, one of the concerns was the loss of a graft-vs.-leukemia effect, given the pharmacological interference with the JAK1/2 signal pathway; however, the reported rate of disease recurrence after Ruxolitinib was of 9.3 and 2.4 % of the patients in the aGVHD and cGVHD groups, respectively. Initial signs of improvement (partial response) of GVHD symptoms were observed 6 days from the start of Ruxolitinib and complete resolution after 3 weeks of treatment. These findings are similar to those reported by Zeiser et al. (median time to response 1.5 weeks, range 1–11). A possible synergistic role with budesonide and MMF cannot be completely ruled out even though these agents were administered only for a very few days. The most effective Ruxolitinib tapering schedule remains to be defined, given the limited clinical experience. Our schedule was purely based on clinical grounds in the light of GvHD signs/symptoms, worsening of cytopenias, or viral reactivation. A steroid-sparing effect in chronic GvHD has also recently been proposed [26] with encouraging results. A similar role in aGvHD has not yet been reported. Ruxolitinib dose-dependent cytopenias are usually expected during treatment [27] even though, in our patient, other factors such as GvHD itself and prolonged immunosuppressive therapy with several agents may have been involved [28]. At 5 months from HSCT, the patient is doing well, with full donor mixed chimerism and in continuous complete remission (confirmed at bone marrow biopsy on day 148) with no signs and/or symptoms of GvHD on CsA taper. Prednisone was stopped on day 135 and Ruxolitinib on day 156. Of note, there was no CMV (though both donor and recipient were CMV seronegative)- nor Epstein Barr Virus-DNAemia breakthrough. Overall, our clinical findings suggest that Ruxolitinib played a fundamental role in the successful GvHD treatment of our patient. SR-GvHD is a challenging complication with no current standard treatment. GI tract involvement is life-threatening also in the light of the long process required for a complete repair of the intestinal mucosa. Therapeutic efficacy of Ruxolitinib is underlined by recent biological insights into the relevant role played by the JAK-STAT signaling pathway in aGvHD. However, treatment duration and possible impacts on graft-vs.-leukemia and immune-responses against infections remain a matter of debate. Thus, larger clinical phase II–III controlled trials and comparisons with best available treatments are warranted. A German multi-center phase II clinical trial on Ruxolitinib in SR-aGvHD is due to start accrual this fall (NCT02396628).

## Abbreviations

aGvHD, acute graft-vs.-host disease; cGvHD, chronic GvHD; CMV, cytomegalovirus; GI, gastrointestinal; HSCT, hematopoietic stem cell transplantation; i.v., intravenous; JAK, Janus kinases; MMF, mycophenolate mofetil; RAEB-1, refractory anemia with excess blast type-1; SR-GvHD, steroid-refractory graft-vs.-host disease; Treg, regulatory T cells
